# An Inexpensive CRISPR-Based Point-of-Care Test for the Identification of Meat Species and Meat Products

**DOI:** 10.3390/genes13050912

**Published:** 2022-05-19

**Authors:** Dagang Tao, Xiao Xiao, Xiaochen Lan, Bingrong Xu, Yuan Wang, Emmanuel Mulaya Khazalwa, Wenya Pan, Jinxue Ruan, Yu Jiang, Xiangdong Liu, Changchun Li, Ruizhen Ye, Xinyun Li, Jing Xu, Shuhong Zhao, Shengsong Xie

**Affiliations:** 1 Key Laboratory of Agricultural Animal Genetics, Breeding and Reproduction of Ministry of Education & Key Lab of Swine Genetics and Breeding of Ministry of Agriculture and Rural Affairs, Huazhong Agricultural University, Wuhan 430070, China; dagangtao@foxmail.com (D.T.); xiaoxiaoz320@foxmail.com (X.X.); llan@axiom-genetics.cn (X.L.); xubingrongxu@163.com (B.X.); wangyuanhzau@foxmail.com (Y.W.); yawenpan@126.com (W.P.); ruanjinxue@mail.hzau.edu.cn (J.R.); liuxiangdong@mail.hzau.edu.cn (X.L.); lichangchun@mail.hzau.edu.cn (C.L.); yrz20011205@163.com (R.Y.); xyli@mail.hzau.edu.cn (X.L.); hzauxujing@163.com (J.X.); shzhao@mail.hazu.edu.cn (S.Z.); 2 Guangdong Laboratory of Lingnan Modern Agriculture, Guangzhou 510642, China; 3 International Livestock Research Institute (ILRI), P.O. Box 30709, Nairobi 00100, Kenya; emmanuelbahati235@gmail.com; 4 Hubei Hongshan Laboratory, Frontiers Science Center for Animal Breeding and Sustainable Production, Wuhan 430070, China; 5 Yangshan Customs, Shanghai 201306, China; gdtyxfk013@163.com

**Keywords:** CRISPR/Cas12a, LAMP, colorimetric, lateral flow detection, NADH4, ND2, D-loop

## Abstract

The growing demand for and supply of meat and meat products has led to a proportional increase in cases of meat adulteration. Adulterated meat poses serious economic and health consequences globally. Current laboratory methods for meat species identification require specialized equipment with limited field applications. This study developed an inexpensive, point-of-care Loop-Mediated Isothermal Amplification (LAMP)-CRISPR/Cas12a colorimetric assay to detect meat species using a Texas Red-labelled single-strand (ssDNA) reporter. As low as 1.0 pg/µL of the porcine *NADH4*, the chicken NADH dehydrogenase subunit *2* (*ND2*) and the duck *D-loop* genes was detectable under white, blue and ultraviolet light. The test turnaround time from DNA extraction to visualization was approximately 40 min. The assay accurately detected pure and mixed-meat products in the laboratory (*n* = 15) and during a pilot point-of-care test (*n* = 8) in a food processing factory. The results are 100% reproducible using lateral flow detection strips and the real-time PCR detection instrument. This technology is fully deployable and usable in any standard room. Thus, our study demonstrates that this method is a straightforward, specific, sensitive, point-of-care test (POCT) adaptable to various outlets such as customs, quarantine units and meat import/export departments.

## 1. Introduction

The accurate identification of animal-derived products is key to monitoring the authenticity of livestock commodities [1]. Meat adulteration is a widespread global problem impacting the food processing trade, resulting in adverse economic losses [2]. Consumers, food regulators, manufacturers and processors are increasingly becoming conscious of animal products. Inaccurate and false labelling of meat and meat products can harm consumer health and religious beliefs [3]; for example, Muslim halal laws prohibit the consumption of any food products containing pork [4].

In recent years, the variation in meat prices has widened due to the impact of the 2019 novel coronavirus (SARS-CoV-2) pandemic [5] and the African swine fever virus (ASFV) epidemic [6]. The use of substandard products by unscrupulous merchants in pursuit of profit has become increasingly common, with opportunities for adulteration increasing as the modern food industry becomes more finely divided and the supply chain broadens [7]. For example, beef and lamb can either be substituted or supplemented with cheaper meat such as pork, chicken and duck to increase the profit margins [8]. Since adulterated meat comes from untrademarked sources, it is neither tested nor inspected by the public health departments. Thus, it may harbour unknown harmful pathogens and contain additives such as colouring and chemicals that might be hazardous to consumers’ health [9]. The use of non-edible meat disguised as edible meat, such as the European horse meat scandal [10] and the Jinan Wal-Mart fox meat instead of donkey meat incident [11], is more dangerous as these animals are reared using veterinary medicines that are likely to remain in the meat products, causing more damage when consumed by humans [12]. Thus, it is essential to establish an effective and sensitive method to detect animal-derived ingredients.

Traditional techniques used to identify meat species include morphological, cytological, biochemical and molecular-based methods. The enzyme-linked immunosorbent assay (ELISA) [13], chromatography (LC and HPLC) [14,15], spectroscopy and electrophoretic assays detect species-specific proteins in animal products. However, these conventional methods are costly, less specific, time-consuming and require skilled personnel. Molecular techniques detect species-specific DNA and are preferred for meat identification because they overcome the limitations of protein-based detection methods such as species specificity [16]. Additionally, molecular methods such as the polymerase chain reaction (PCR) can be multiplexed and modified (e.g., RAPD-PCR, RFLP-PCR, real-time-PCR) to meet specific needs [16]. The major drawback of PCR is that it requires sophisticated instruments and trained operators and hence is not suitable for field-based analyses.

Presently, isothermal amplification [17,18,19] and CRISPR detection technologies [20,21,22] are rapidly developing in the field of nucleic acid diagnostics. Species-specific meat detection systems using CRISPR/Cas12a technology and DNA barcoding strategies are available [23]. Liu et al., 2020, developed a method based on recombinase polymerase amplification (RPA) and CRISPR/Cas12a technology to detect food-derived pathogenic bacteria and food adulteration [24]. These methods are fast and specific but require complex detection instruments, limiting their applications in field testing. There is an urgent need for a point-of-care test method for meat species identification and authentication adaptable to customs, animal quarantine units and meat import/export departments. In this study, we employed Loop-Mediated Isothermal Amplification (LAMP) and CRISPR/Cas12a cleavage with Texas Red-labelled single-strand (ssDNA) reporters to develop an inexpensive, point-of-care colorimetric assay for the detection and identification of meat species in meat and meat products.

## 2. Materials and Methods

### 2.1. Sample Preparation

Verified meat from bovine, canine, chicken, duck, equine, ovine and porcine species was purchased from the local licenced supermarket in Wuhan, China. Approximately 0.05 g of the meat samples was placed in sterile 1.5 mL microcentrifuge tubes and stored at −80 °C for further processing. Genomic DNA (gDNA) was extracted from the meat samples using the Blood/Cell/Tissue Genomic DNA Extraction Kit (Cat No: 4992254, TIANGEN) and the QuickExtract™ DNA Extraction Solution (Cat No: QE09050, Lucigen, WI, USA) according to the manufacturer’s protocol. The extracted DNA concentration was determined using the NanoDrop^TM^ 2000 spectrophotometer (Thermo Fisher Scientific, MA, USA).

### 2.2. PCR and LAMP Primers Design

The mitochondrial DNA (mtDNA) of porcine *NADH4*, chicken *ND2* and duck *D-Loop* genes were selected to differentiate inter-specific gene sequences [25,26]. Specific PCR primers (Table S1) targeting the mtDNA in pork, chicken and duck were designed using the “Primer-BLAST” online software (https://www.ncbi.nlm.nih.gov/tools/primer-blast/, accessed on 20 October 2020). Six LAMP primers (Table S2) targeting mtDNA for the *NADH4* (pig), *ND2* (chicken) and *D-loop* (duck) genes were designed by Primer Explorer V5 (http://primerexplorer.jp/lampv5e/, accessed 25 October 2020).

### 2.3. Species-Specific CRISPR RNA (crRNAs) Design

Five crRNAs for each species (Table S3) targeting the *NADH4* (pig), *ND2* (chicken) and *D-loop* (duck) genes with a 5′ TTTN protospacer-adjacent motif (PAM) in the DNA strand opposite the target sequence were designed using CRISPR-offinder (www.biootools.com, accessed 22 October 2020) [27]. The crRNA and single-strand (ssDNA) reporter (Table S3) were synthesized by Tsingke Biological Technology (Beijing, China).

### 2.4. Assessment of crRNAs and LAMP Primers for Intra-Specific Conservation

FASTA files of target mtDNA for pigs, chicken and duck were downloaded from the National Center for Biotechnology Information (NCBI) database (https://www.ncbi.nlm.nih.gov/, accessed 10 October 2020) (Table S4). Candidate LAMP and crRNA sequences were evaluated using the online Clustal Omega multiple sequence alignment software (https://www.genome.jp/tools-bin/clustalw, accessed 25 October 2020) (Figure S1).

### 2.5. In Vitro RNA Transcription Using T7 RNA Polymerase

crRNA targeting pig *NADH4*, chicken *ND2* and duck *D-loop* genes were transcripted from pUC57-T7-crRNA plasmids and amplified by PCR using a forward primer containing the T7 promoter, a reverse primer containing the nucleotide target sequences (Table S1) and Premix Taq (Cat No: RR902A, TaKaRa, Beijing China). The PCR amplicons were purified using the PCR purification kit (Cat No: D4001S, Tianmo Sci&Tech Development, Beijing, China) and transcribed using the HiScribe T7 High Yield RNA Synthesis Kit (Cat No: E2040S, NEB, Ipswich, MA, USA). In vitro-transcribed RNA was purified using the Monarch RNA Cleanup Kit (Cat No: T2040S, NEB, Ipswich, MA, USA). The RNA quality and quantity were evaluated on a 2% agarose gel and the NanoDrop^TM^ 2000 spectrophotometer (Thermo Fisher Scientific, Waltham, MA, USA), respectively.

### 2.6. LAMP-CRISPR/Cas12a Fluorescence Assay

DNA templates were LAMP-amplified in a 25 μL reaction volume containing 2.5 µL of 10 × Isothermal Amplification Buffer II, 6 mM MgSO_4_ (B1003S, NEB), 14 mM dNTP Mix (Cat No: N0447S, NEB, MA, USA), 1.0 µL of Bst 3.0 DNA Polymerase (Cat No: M0374L, NEB, MA, USA), 1.6 μM of FIP and BIP, 0.2 μM F3 and B3, 0.4 μM LF and LB, 1.0 µL of DNA template, 0.5 µL of Uracil-DNA Glycosylase (UDG, Cat No: M0280S, NEB, MA, USA), 0.2 mM dUTP (Cat No: N0459S, NEB, MA, USA) and DEPC (Diethylpyrocarbonate)-treated water. Aerosol formation was minimized by adding 0.5 μL of UDG and 0.2 mM dUTP over the reaction solution. Reagents were prewarmed at 37 °C for 5 min, and the LAMP reaction was conducted in a heat block at 65 °C for 40 min, followed by a termination step at 98 °C for 2 min. A total of 4 µL of the LAMP products was taken and visualized on a 2% agarose gel.

Next, 2 μL of preamplified products was added to CRISPR/Cas12a detection mixture (250 nM Cas12a (Cat No: M0653T, NEB, MA, USA), 500 nM crRNA, 1.5 µM ssDNA reporter (JOE-labelled reporter) and 2 µL of NEB buffer 2.1 (Cat No: B7203S, NEB, MA, USA) and incubated at 37 °C for 10 min. Fluorescence was observed with the naked eye using a portable blue-light transilluminator (BioTeke Co., Wuxi, China) and a mini UV gel imager (Peiqing Technology Co., Shanghai, China), and images were captured using a smartphone. The fluorescence signal changes were recorded using the Applied Biosystems^TM^ 7500 real-time PCR System (Cat No: 4351105, ThermoFisher Scientific, MA, USA) set at 37 °C for 99 cycles. The fluorescence signals were collected every 90 s (per cycle). The fluorescence intensity was read using the EnSpire Multimode microplate reader (PerkinElmer, Akron, OH, USA).

### 2.7. LAMP-CRISPR/Cas12a Colorimetric Assay

LAMP amplification was performed as detailed in Section 2.6. However, the Cas12a digestion of the LAMP products incorporated a Texas Red-modified reporter (Table S3). The Cas12a enzymatic digestion reaction system contained 500 nM Cas12a, 1 µM crRNA, 10 µM/2.5 µM ssDNA reporter, 2 µL of NEB buffer 2.1 and 2 µL of preamplified products. The Cas12a conditions involved an initial incubation at 37 °C for 10 min and terminated at 98 °C for 2 min. The reaction tubes were visualized with the naked eye under white light, blue light and ultraviolet light with immediate photography.

### 2.8. Sensitivity and Specificity of the LAMP-CRISPR/Cas12a Colorimetric Assay

The sensitivity of the LAMP-CRISPR/Cas12a colorimetric assay was tested using 10-fold dilutions (1 ng–0.1 pg) of the verified DNA. Bovine, canine, chicken, duck, equine, ovine and porcine gDNA quality was verified using species-specific PCR primers (Table S1, Figure S4).

The assay specificity was determined using gDNA extracted from species or a mock preparation of fifteen mixed-meat products (Table S5) containing pork, chicken, duck, cattle, sheep, horse and dog meat. LAMP-CRISPR/Cas12a assays combined with these fifteen models with different mixing ratios were compared and analyzed with the naked eye under white light, blue light and ultraviolet light with immediate photography. The endpoint fluorescence signal intensity was analyzed using the EnSpire Multimode microplate reader (PerkinElmer, Akron, OH, USA).

### 2.9. LAMP-CRISPR/Cas12a with Lateral Flow Dipstick (LFD) Assay

The LAMP products were cleaved using the Cas12a enzyme with an FAM-N_12_-Biotin modified reporter (Table S3). The optimal parameters for CRISPR/Cas12a detection system were as follows: 2 μL of 10 × NEB Buffer 2.1, 500 nM Cas12a, 1 μM crRNA and 1.5 µM ssDNA reporter, topped to 20 μL with sterile ultrapure water. The setup was incubated at 37 °C for 30 min and then at 98 °C for 2 min. The Cas12a cleavage products were transferred into a 1.5 mL microcentrifuge tube containing 80 µL of Milenia Gen line Dipstick Assay Buffer from HybriDetect─Universal Lateral Flow Assay Kit (Milenia Biotec, Gießen, Germany) and mixed for 5 min at room temperature using a gentle stream of air. Test bands were observed on a lateral flow strip paper with a streptavidin conjugate pad.

### 2.10. On-Site Application of the LAMP-CRISPR/Cas12a Assay

We developed an on-site nucleic acid detection toolkit for visual identification of pork, chicken and duck in meat products with the LAMP-CRISPR/Cas12a assay. The nucleic acid detection toolkit included a portable UV flashlight, 100/10 μL pipettes, pipette tips, a dry bath and a mini centrifuge. Reagents such as the QuickExtract DNA Extraction Solution (Cat No: QE09050, Lucigen, Middleton, WI, USA), the LAMP mixtures (1 μL of Bst 3.0 DNA polymerase, 2.5 μL of 10× isothermal amplification buffer, 6 mM MgSO_4_, 14 mM each of dNTP Mix and 2.5 μL of primer mix) and CRISPR/Cas12a mixtures (250 nM Cas12a, 500 nM crRNA, 1.5 µM ssDNA reporter, 2 µL of NEB buffer 2.1) were transported in dry ice. Quick gDNA extraction was achieved by adding the meat samples to 20 μL of Lucigen QuickExtract DNA Solution and incubating at 95 °C for 5 min. A total of 2 μL of the reaction product was added into the LAMP reaction and incubated at 65 °C for 25 min. Then, 2 μL of the LAMP amplicons were assayed using the Cas12a detection assay and incubated at 37 °C for 10 min. Visual colorimetric/fluorescence read-outs were analyzed by naked eye and a portable UV flashlight. All images were processed and analyzed using the Adobe Illustrator 2020 and Adobe Photoshop CC 2019 software.

## 3. Results

### 3.1. CRISPR/Cas12a crRNA Activity

**Figure 1:**
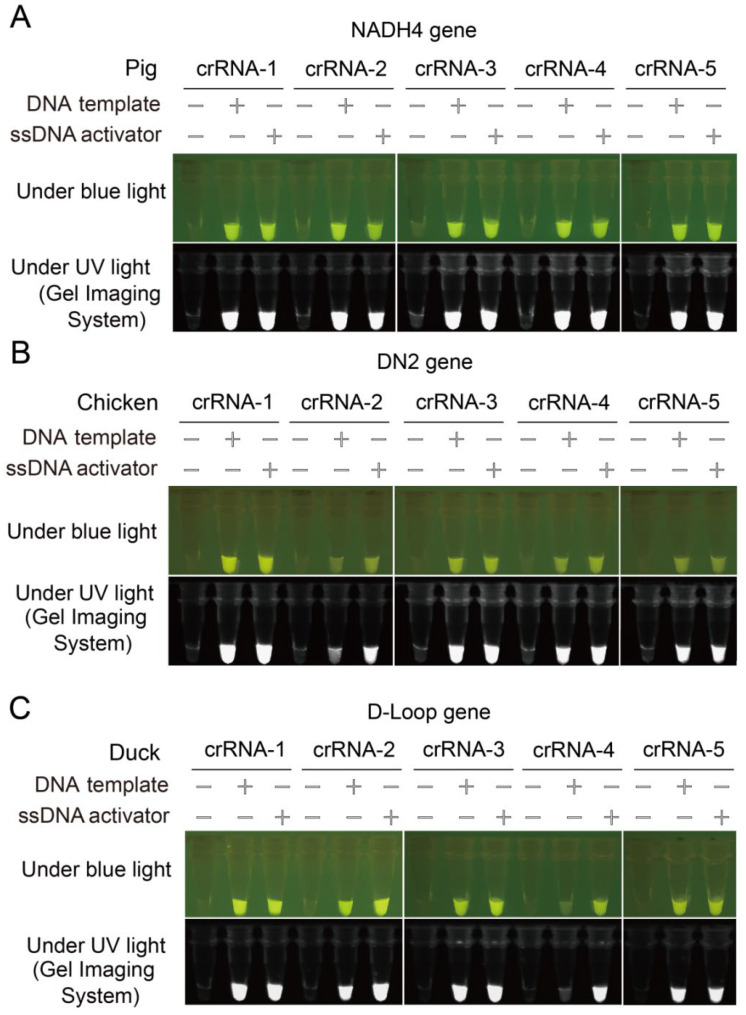
Identification of the highly active crRNAs for detection of pork, chicken and duck meat using CRISPR-Cas12a fluorescence assay. Identification of highly active crRNAs for visual detection of (**A**) the pork *NADH4* gene, (**B**) the chicken *ND2* gene and (**C**) the duck *D-Loop* gene. crRNA–1, crRNA–2, crRNA–3, crRNA–4 and crRNA–5 represent different crRNAs targeting the *NADH4*, *DN2* and *D-Loop* genes. DNA templates represent the PCR amplicons of *NADH4, DN2* or *D-Loop* genes. ssDNA activator is a crRNA-complementary ssDNA. Images captured using a smartphone camera under blue (470 nM) and ultraviolet lights.

Highly active crRNAs are needed to successfully establish CRISPR/Cas12a-based on-site nucleic acid detection systems. The trans-cleavage activity of fifteen selected crRNAs targeting the pig *NADH4*, the chicken *ND2* and the duck *D-loop* genes was evaluated (Figure 1). Five crRNAs targeting the porcine *NADH4* gene were highly active with strong fluorescence signals (Figure 1A). Varying degrees of cleavage activity were observed with the crRNAs targeting the chicken mitochondrial *ND2* gene, with the maximum fluorescence intensity seen in Chicken-crRNA-1, then Chicken-crRNA-3, Chicken-crRNA-5, Chicken-crRNA-4, and Chicken-crRNA-2 (Figure 1B). Four crRNAs targeting the duck *D-loop* gene were highly active except for Duck-crRNA-4 (Figure 1C). The Pig-crRNA-5, the Chicken-crRNA-1 and the Duck-crRNA-1 were selected to develop the CRISPR/Cas12a-based nucleic acid detection assay in the subsequent experiments.

### 3.2. Sensitivity Evaluation of the LAMP-CRISPR/Cas12a Fluorescence Assay

Two sets of LAMP primers for each animal species targeting segments near the crRNA binding site were designed (Figure S1) and analyzed by gel electrophoresis (Figure S2). DNA bands were visible with the porcine LAMP-pig-2 (Figure S2A), the chicken LAMP-ch-1 (Figure S2) and the duck LAMP-du-1 and LAMP-du-2 (Figure S2C) primers. The duck LAMP-du-1 primer resulted in brighter and well-defined DNA bands. Downstream analyses used the LAMP-pig-2, LAMP-ch-1 and LAMP-du-1 sets of primers.

**Figure 2:**
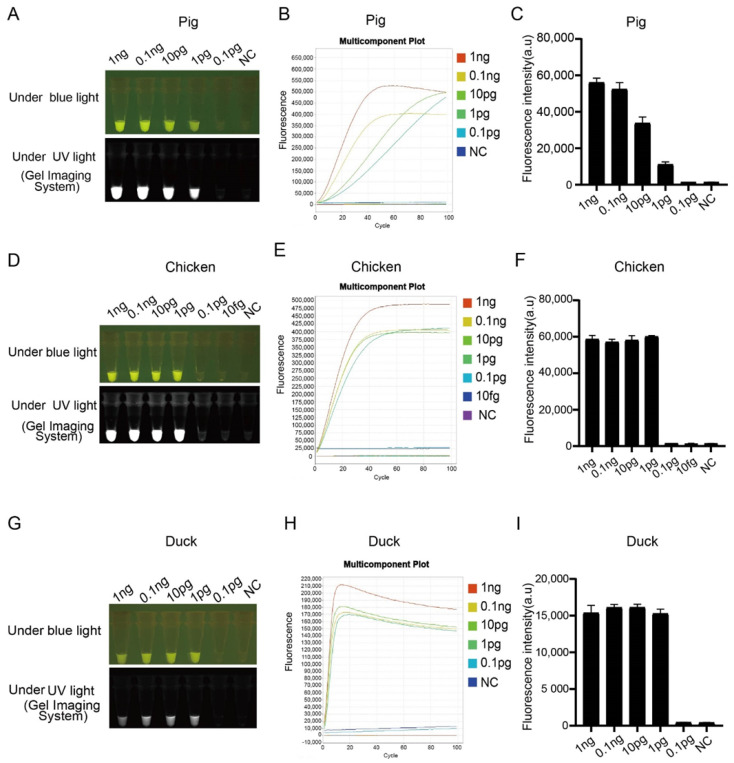
Assessing the sensitivity of LAMP combined with CRISPR/Cas12a technology for pork, chicken and duck meat detection. (**A**,**D**,**G**) Sensitivity of LAMP combined with CRISPR/Cas12a technique to visually detect pork, chicken and duck under blue and ultraviolet lights; (**B**,**E**,**H**) detection of fluorescence using a real-time PCR machine; (**C**,**F**,**I**) the end-point signal intensity measured using a fluorescence microplate reader. Data are represented as means ± SEM; *n* = 3. NC stands for negative control.

The sensitivity of the LAMP-CRISPR/Cas12a-based fluorescence assay was evaluated using a dilution gradient of the pork, chicken and duck gDNA. The *NADH4*, *ND2* and *D-Loop* genes were LAMP-amplified (Figure S3), products digested by Cas12a and subjected to fluorescence detection under blue and UV light (Figure 2). Fluorescence as low as 1.0 pg of DNA was detected with the naked eye (Figure 2A,D,G) and with the quantitative PCR machine (Figure 2B,E,H). These were consistent with the end-point fluorescence signal intensity evaluated by a fluorescence microplate reader (Figure 2C,F,I).

### 3.3. Establishment and Specificity Assessment of the LAMP-CRISPR/Cas12a Colorimetric Meat Detection Assay

**Figure 3:**
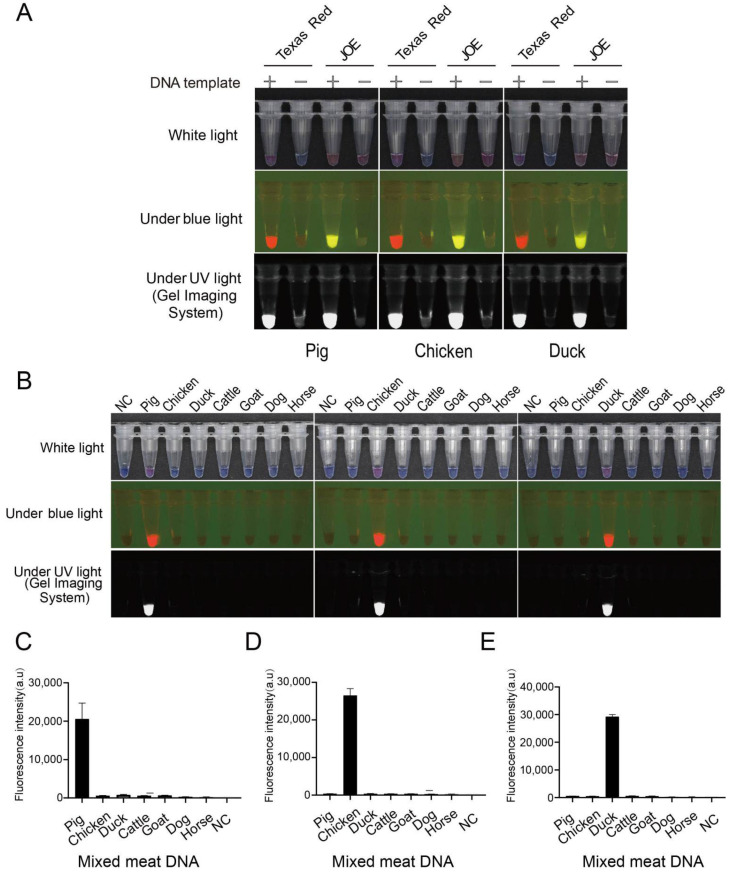
Establishing a naked-eye colorimetric CRISPR/Cas12a assay and assessing the specificity of pork, chicken and duck meat detection. (**A**) Comparison of colorimetric and fluorescence signal of JOE- and Texas Red-labelled ssDNA reporters. (**B**) Assessment of the specificity of LAMP combined with a naked-eye colorimetric CRISPR/Cas12a technology. (**C**–**E**) The end-point signal intensity from Figure 3B was further measured using a fluorescence microplate reader. DNA template represents LAMP amplification of *NADH4, DN2* or *D-Loop* genes. Texas Red stands for Texas Red-labelled ssDNA reporter, JOE stand for JOE-labelled ssDNA reporter; and NC stands for negative control. Data are represented as means ± SEM; *n* = 3.

To simplify the detection process, we compared the capacity of the JOE-modified and the Texas Red-labelled ssDNA reporters for naked-eye colorimetric nucleic acid detection (Figure 3). Although both ssDNA reporters differentiated positive and negative test results under fluorescent conditions, only the Texas Red-labelled ssDNA reporter was perceptible with the naked eye under white light (Figure 3A). Thus, Texas red-labelled ssDNA reporters were preferred to devise the assay. Bovine, equine, ovine and dog gDNA was used to verify the specificity of the LAMP-CRISPR/Cas12a colorimetric technology detection of pork, chicken and duck meat. First, species-specific DNA quality was verified using universal 18s *rRNA* and species-specific primers. DNA fragments of the expected band size were obtained (Figure S4A–D). Additionally, species-specific genes were LAMP-amplified from these verified gDNAs. The amplicons were subjected to CRISPR/Cas12a digestion using the Texas Red-labelled ssDNA reporter for detection. Colorimetric and fluorescence readouts under white, blue and UV lights were observable in reactions containing gDNA samples from the target species (Figure 3B). This specificity was consistent with the fluorescence intensity on the microplate reader (Figure 3C–E). Therefore, the LAMP-CRISPR/Cas12a colorimetric assay had high specificity for accurately distinguishing the DNA of pork, chicken and duck meat components.

### 3.4. Colorimetric and Lateral Flow Detection in Mixed-Meat Products

**Figure 4:**
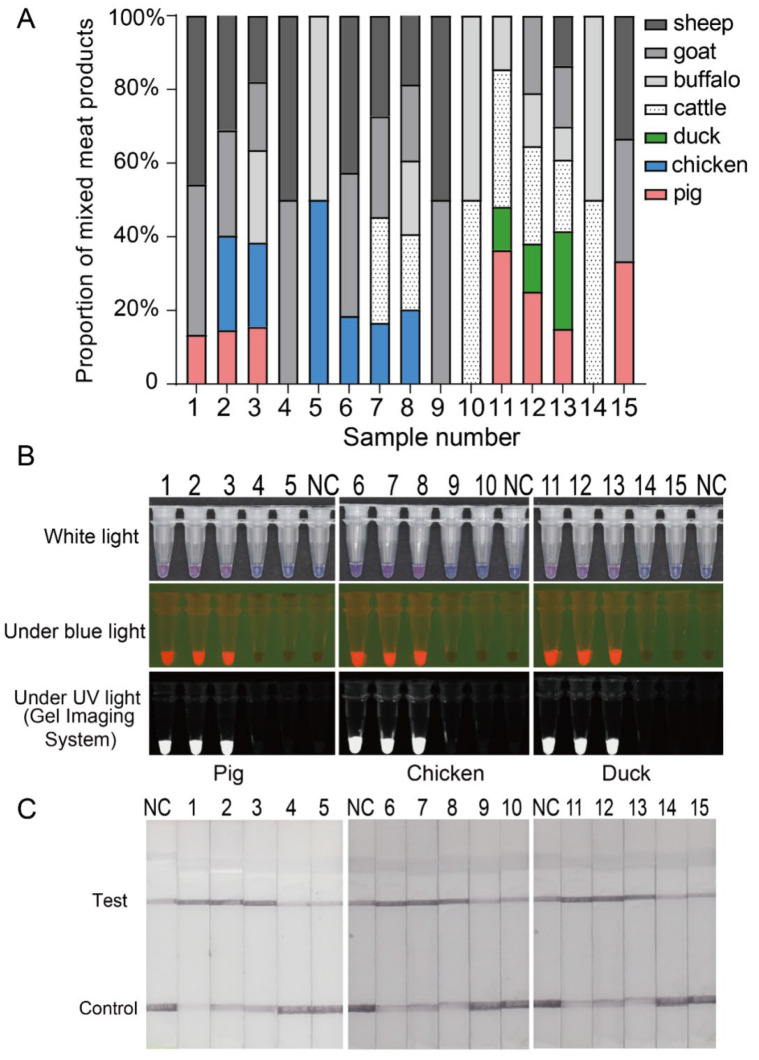
Detection of pork, chicken, and duck in simulated mixed meats based on naked-eyed colorimetric CRISPR/Cas12a assay. (**A**) Proportion of animal-derived components in simulated mixed meat samples. (**B**) Identification of pork, chicken and duck in simulated mixed meats based on naked-eyed colorimetric CRISPR/Cas12a assay. (**C**) Identification of pork, chicken and duck in simulated mixed meats based on CRISPR/Cas12a assay combined with a lateral-flow paper strip. 1–15 represents simulated mixed-meat samples. NC stands for negative control.

To evaluate the application of the naked-eye colorimetric detection of pork, chicken and duck in mixed-meat samples, 15 conditions of mixed-meat products with different mix ratios (binary, ternary, quaternary and quintuple) were tested (Figure 4A, Table S5). Colour changes and fluorescence discernible to the naked eye under white, blue and ultraviolet lights (Figure 4B) were observed only in mixed-meat products containing the target species; pork (samples 1, 2, 3), chicken (samples 6, 7, 8) and duck (samples 11–13). The negative controls and mixed-meat products devoid of pork (samples 4, 5), chicken (samples 9, 10),and duck (samples 14, 15) meat did not exhibit any colorimetric and fluorescent read-outs. These results were 100% consistent with the mixed-meat identification by CRISPR/Cas12a combined with a lateral-flow paper strip assay (Figure 4C).

### 3.5. On-Site Application of the CRISPR/Cas12a Colorimetric Assay

**Figure 5:**
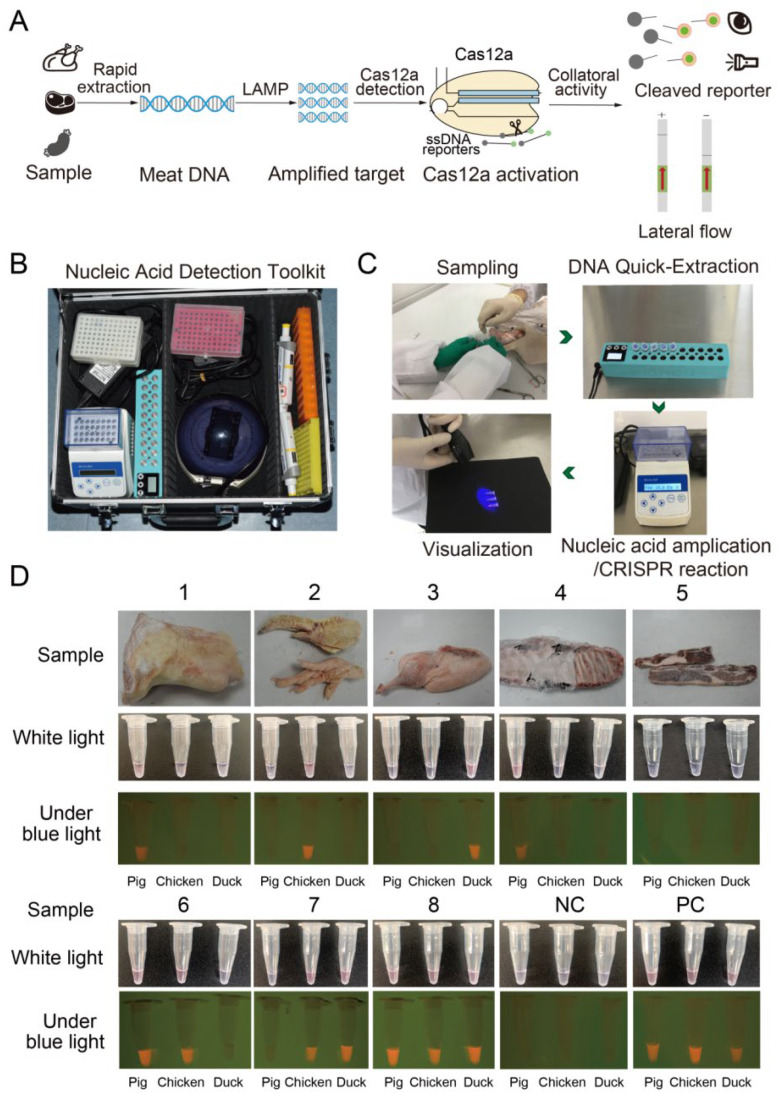
One-site application of the naked-eye colorimetric CRISPR/Cas12a assay for meat detection and identification. (**A**) Schematic diagram in the CRISPR/Cas12a assay for the detection of pork, chicken and duck. (**B**) Portable nucleic acid detection toolkit. (**C**) On-site nucleic acid detection steps of meat identification. (**D**) The results of meat identification in 8 samples. Samples 1–5 represent random-test meat samples, 6–8 represent mixed-meat samples. PC stands for positive control. NC stands for negative control.

To truly get rid of the laboratory and realize on-site species identification of meat, we developed a process for point-of-care detection based on the visual LAMP-CRISPR/Cas12a colorimetric assay (Figure 5A). We first devised a portable nucleic acid detection toolkit to identify pork, chicken and duck meat outside the laboratory. The toolkit contained a portable heater, a mini centrifuge and pipettes (Figure 5B). We then conducted an on-site meat identification test in a standard room at a local food processing factory involving sampling, quick DNA extraction, amplification and CRISPR/Cas12a digestion steps (Figure 5C) to identify pork, chicken and duck meat. A total of eight blinded meat samples (single and mixed) were tested and accurately identified by the naked-eye meat identification colorimetric assay (Figure 5D). We accurately identified samples 1, 2 and 3 as pork, chicken and duck meat, respectively (Figure 5D). Sample 6 was a mixture of pork and chicken, and sample 7 was a mixture of chicken and duck meat, while sample 8 comprised pork, chicken and duck meat (Figure 5D). Sample 4 was pork while sample 5 did not have pork, chicken or duck meat. Therefore, we successfully applied the naked-eye colorimetric CRISPR/Cas12a assay in the field to detect pork, chicken and duck outside the laboratory.

## 4. Discussion

This study established an inexpensive, rapid, naked-eye visualization method based on the CRISPR/Cas12 system to detect meat species. As an integral part of the human diet, the safety of meat and meat products is becoming more prominent. Meat adulteration continues to impact the food safety systems and has a negative economic impact on society, posing a serious threat to consumer health. The development of a fast, simple and accurate detection technology for meat-derived components is urgent due to the large circulation of meat products and their short shelf life at room temperature [28]. Presently, fluorescence-based nucleic acid detection methods are used in pathogen identification [29,30,31]. This study combined LAMP, CRISPR/Cas12a crRNA targeting with Texas Red-labelled ssDNA reporter to develop a simple, sensitive and specific point-of-care colorimetric detection system discernible to the naked eye under white, blue and ultraviolet light. DNA from meat and meat products was first isothermally amplified and cleaved by CRISPR/Cas12a activity detectable with ssDNA reporters such as the Texas Red-labelled ssDNA reporter.

Low copy numbers of animal nuclear DNA (nDNA) [32] limit the sensitivity of nDNA-based assays. In contrast, mtDNA has significant thermal stability and a high copy number [33]. Mitochondrial genes such as *D-loop* [34], *Cytb* [35,36] and 12S *rRNA* [37,38,39] are universally used for meat product composition identification. We conducted interspecies-specific and species-conservative comparisons of mitochondria endogenous genes in different porcine, chicken and duck breeds. The endogenous reference genes *NADH4* (porcine), *DN2* (chicken) and *D-loop* (duck) were identified and evaluated (Figure S1). LAMP primers for each endogenous gene fragment were designed and used to amplify species-specific nucleic acids. Moreover, crRNAs were designed for each target gene for Cas12a cleavage activity. Changes in colour and fluorescence intensity were used to characterize the sample composition.

The LAMP-CRISPR/Cas12a colorimetric assay is highly sensitive, exhibiting a minimum LoD of 1.0 pg of gDNA for all the three animal species selected for this study. This was detectable with the naked eye under white, blue and ultraviolet light with high fluorescence intensity. Correspondingly, this was the detection limit of the advanced real-time PCR fluorescence visualization instrument [16]. Therefore, the assay without complex visualization instruments is reliable for application in conditions with low DNA copy numbers.

The assay’s specificity was evaluated using mixed-meat products. The test accurately detected our target meat samples from fifteen different laboratory simulations of mixed-meat products. We also selected a food processing factory in the field where we were able to detect the target species in randomly selected pure and mixed-meat samples. This was the first point-of-care application of the assay in a non-laboratory environment with an excellent performance in detecting meat species. The detection was purely conducted with the naked eye under white and blue light. The results are reproducible using lateral flow detection strip papers, providing individuals with colour impairment an alternative method of detection.

This assay is easy to perform and has a shorter turnaround time compared to traditional chromatography and immunoassays used for meat species detection. It is inexpensive as ssDNA reporters are easy to prepare and stable for long periods [40]. The visual colour is concentration-dependent, making it suitable for samples with low DNA concentrations. In addition, the method does not require expensive equipment, except for a small heater and a hand-held centrifuge, making it possible to deploy in resource-limited, rural, law enforcement inspection and quarantine facilities. Despite its versatility, this method is purely qualitative; hence, result interpretations might be subjective due to interpersonal perception of colour changes. Thus, qPCR remains an irreplaceable and useful quantitative tool for the identification of meat products. Importantly, compared with qPCR technology, CRISPR-based nucleic acid detection methods can cost less than USD 1 per reaction for meat product identification, while qPCR can cost as much as USD 5. While multiplex PCR can enable the identification of several unknown components, the detection throughput cannot be increased indefinitely due to its non-specific amplification risk and cross-reactivity. To sum up, the development of assay tools for the on-site, visual, high-throughput and quantitative identification of meat product components still needs further exploration.

## Data Availability

Not applicable.

## Supplementary Materials

The following supporting information can be downloaded at: www.mdpi.com/article/10.3390/genes13050912/s1. Figure S1: Sequence alignment of partial sequences of *NADH4*, *DN2* and *D-loop* genes among different pig, chicken and duck breeds, respectively; Figure S2: Screening of LAMP primers targeting porcine, chicken and duck mitochondrial genes; Figure S3: Agarose gel electrophoresis determination of the limit of detection for LAMP amplification; Figure S4: Agarose gel electrophoresis of species-specific PCR. Table S1: PCR amplification primers used in this study; Table S2: LAMP amplification primers used in this study; Table S3: ssDNA-reporters and crRNAs used in this study; Table S4: Species Information; Table S5: Simulate mixed meat products ingredients.
