# Supplementary material for: An Inexpensive CRISPR-Based Point-of-Care Test for the Identification of Meat Species and Meat Products
Source: Genes (Basel). 2022 May 19;13(5):912. doi: 10.3390/genes13050912 (PMC9141687; doi:10.3390/genes13050912)
Supplement: Supplementary file 1 [file genes-13-00912-s001.zip › genes-1679765-suppl.pdf]

Supplementary Information

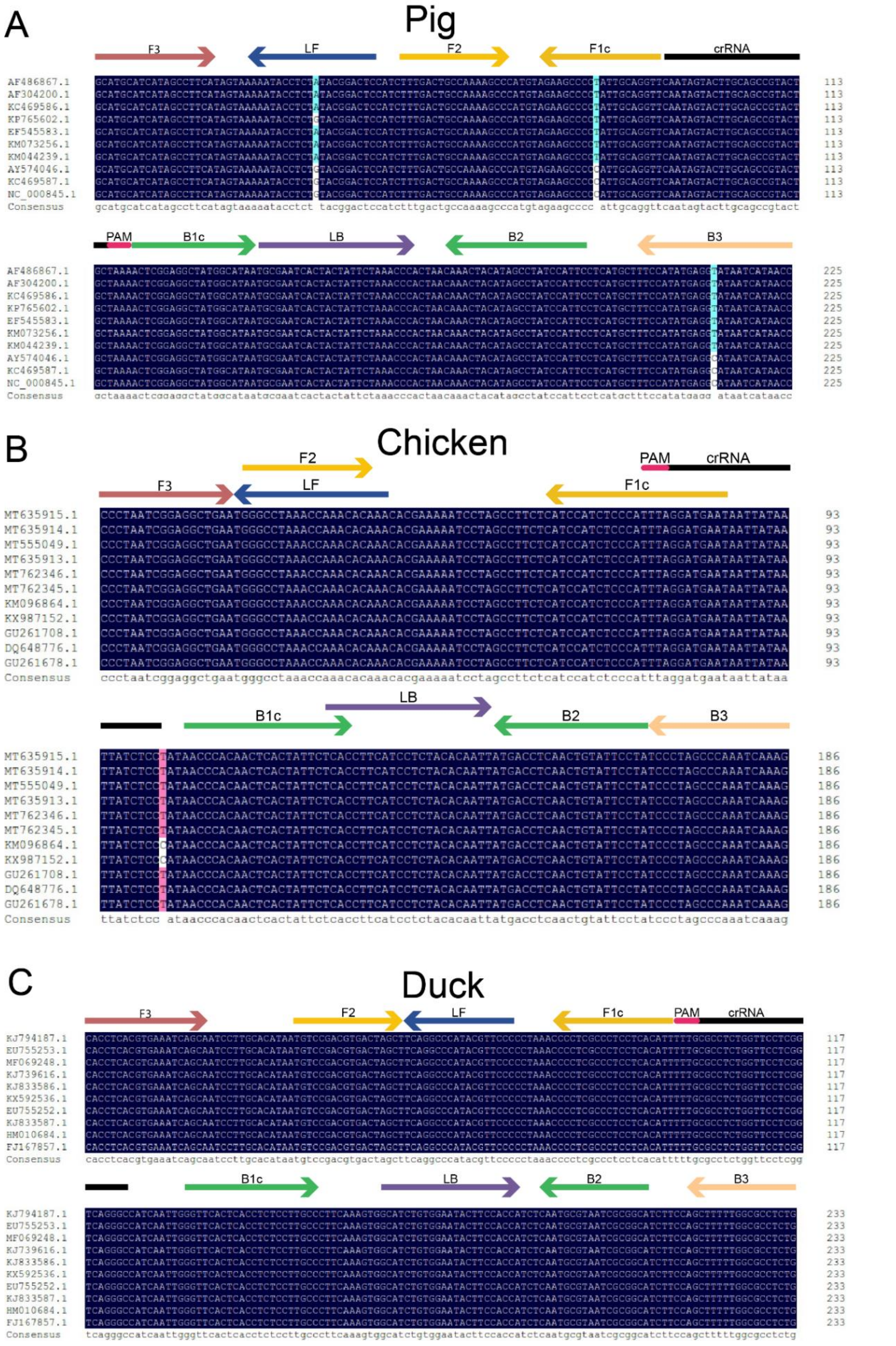

Figure S1. Sequence alignment of partial sequences of NADH4, DN2 and D-loop genes among different pig, chicken and duck breeds, respectively. (A) pig breeds: Chinese Wuzhishan (AF486867.1), Chinese Meishan (AF304200.1), Chinese Jinhua (KC469586.1), Berkshire (KP765602.1), Bamei (EF545583.1), Tibetan (KM073256.1), Rongchang (KM044239.1), Hampshire (AY574046.1), pietrain

(KC469587.1) and the Landrace (NC_000845.1). **(B)** Chicken breeds: Niya (MT635915.1), Longsheng (MT635914.1), Luhua (MT555049.1), Qingyuan (MT635913.1), Wanbei game (MT762345.1), Huangshan black (MT762346.1), Wuhua three-yellow (KM096864.1), Zhengyang Yellow (KX987152.1), Red jungle fowl (GU261708.1), Tibetan (DQ648776.1), and Gushi (GU261678.1). **(C)** Duck breeds: Lin Wu (KJ794187.1), Mallard (EU755253.1), Jinding (MF069248.1), Longsheng (KJ739616.1), Xilin (KJ833586.1), Sichuan (KX592536.1), Pekin (EU755252.1), Rongshui (KJ833587.1), Shaoxing (HM010684.1) and Jianchang (FJ167857.1).

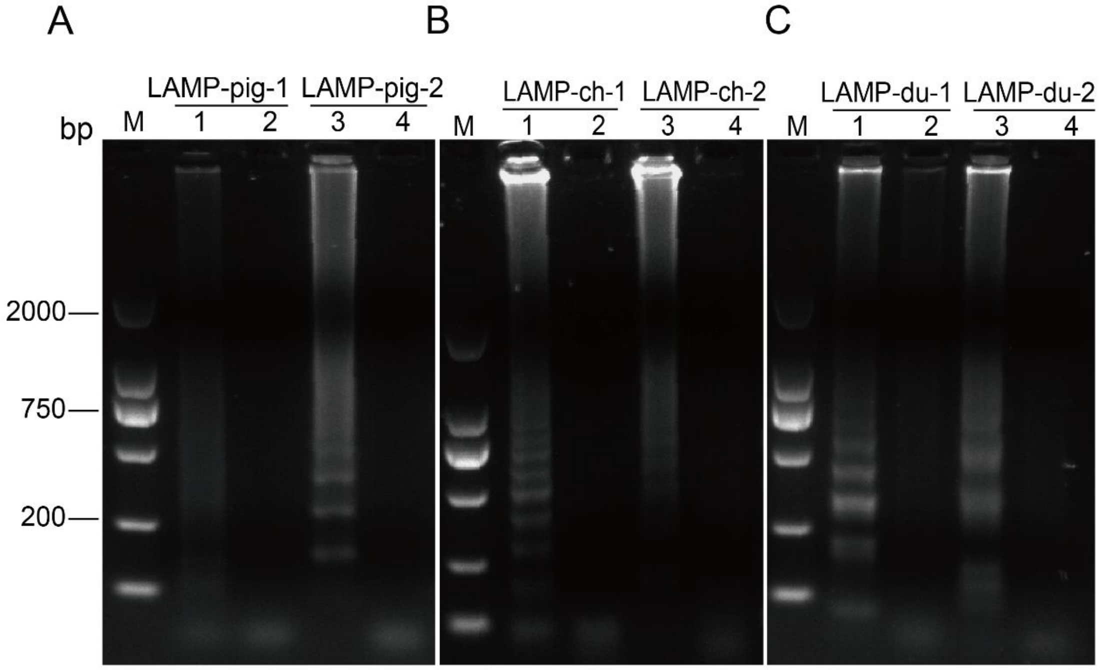

Figure S2. Screening of LAMP primers targeting porcine, chicken and duck mitochondrial genes. Screening of LAMP primers targeting the porcine NADH4, the chicken *ND2* gene and the duck *D-Loop* gene (**A, B, C**).

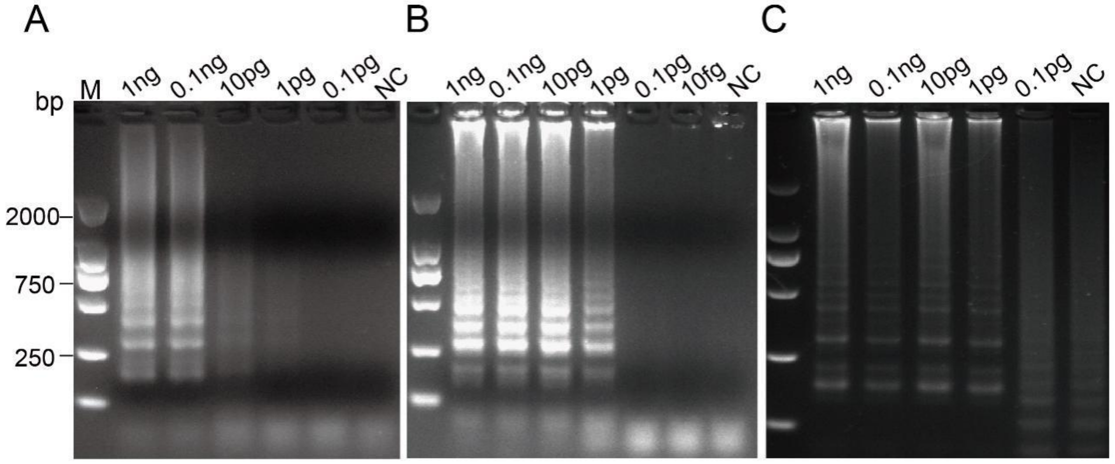

Figure S3. Agarose gel electrophoresis determination of the limit of detection for LAMP amplification. Agarose gel electrophoresis determination of the limit of detection for LAMP amplification of the porcine *NADH4*, the chicken *ND2* and the duck *D-Loop* genes. NC stands for negative control (**A, B, C**).

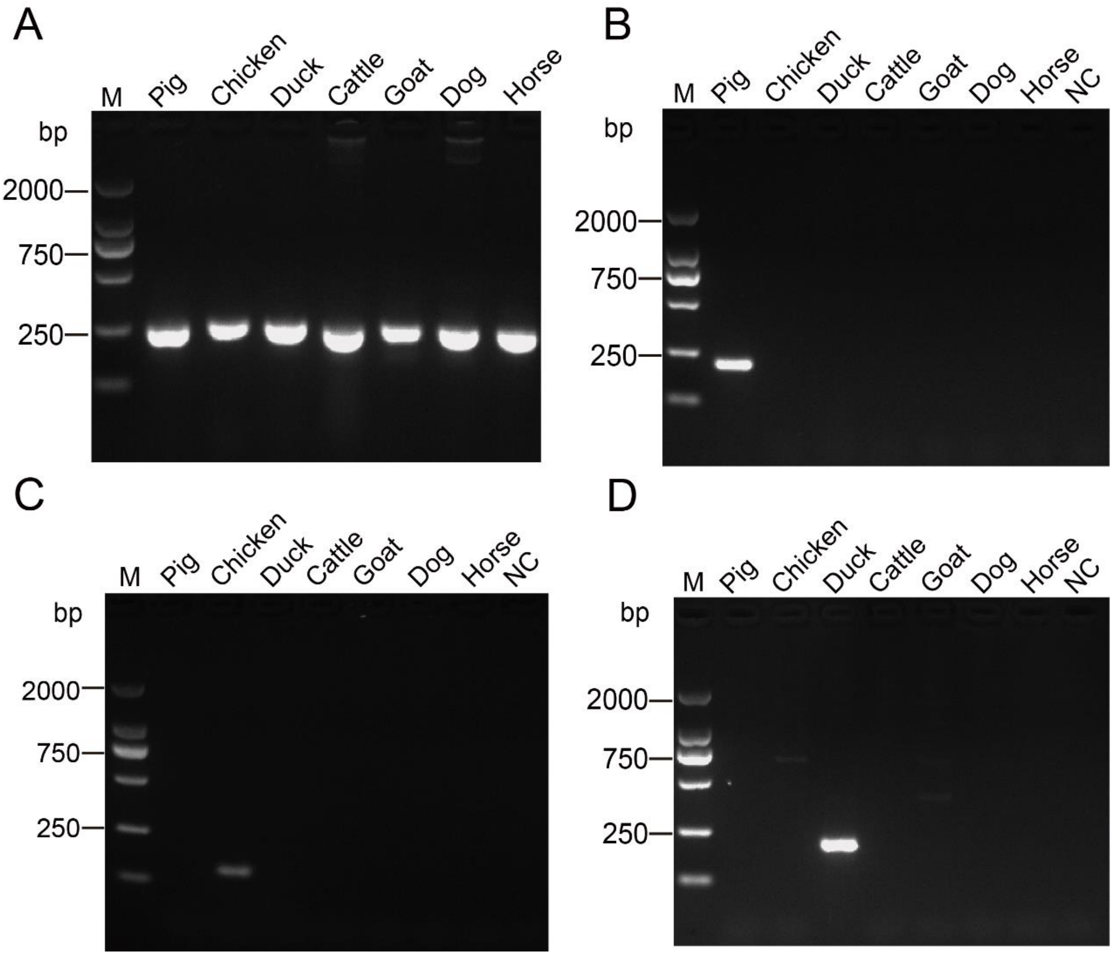

Figure S4. Agarose gel electrophoresis of species-specific PCR. (**A**) Electrophoresis of PCR-amplified products with porcine-specific primers. (**B**) Electrophoresis of chicken-specific primer PCR amplification products. (**C**) Electrophoresis of PCR-amplified products of duck-specific primers. (**D**) Amplification of animal genome by 18s *rRNA* universal primers. NC stands for negative control.

Table S1: PCR amplification primers used in this study

Name	Sequences (5'-3')
Pig-1-F	ATCCTGACGCATACACAGCA
Pig-1-R	ATGGGTGAAGTGGCGTCTTG
Pig-2-F	CGATATGGCCTTTCCACGTA
Pig-2-R	TGTGGGAGATTATTCCGAACCC
Pig-3-F	GCCTAAATCTCCCCTCAATGCTA
Pig-3-R	ATGAAAGAGGCAAATAGATTTTCG
Pig-4-F	CCTACATGCAAACGGAGCAT
Pig-4-R	AGGGTTGTTGGATCCGGTTT
pig-5-F	ACTGATCCCACCCATTATCCAAC
pig-5-R	ATGGTTCGGCTGTGTACTCG
Chicken-1-F	TGCCCTACTACTCTCCACCC
Chicken-1-R	GGTGGGGGTGTTTAGGGTTT
Chicken-2-F	TAGCTGGAATACCCCGACGA
Chicken-2-R	CTAGTGCGACTATCAGGGCG
Chicken-3-F	ACCTACTATACCTGCGGGCAA
Chicken-3-R	ATCCCAGTTTGGGTCCTAGC
Chicken-4-F	TAGAATATGCCGCCGGACCA
Chicken-4-R	GGTATGGGCCCGATAGCTTAG
Chicken-5-F	CCTGAATGCAAATCAGACGCT
Chicken-5-R	GCATGGGCTGTGACGATTAC
Duck-Dloop-F	TCCTCTCCACCCACCCATTA
Duck-Dloop-R	CCCATATACGCCAACCGTCT
18s-F	AGCCTGAGAAACGGCTACC
18s-R	TGCTGGCACCAGACTTGC
pig-PCR-F	GCCTAAATCTCCCCTCAATGCTA
pig-PCR-R	ATGAAAGAGGCAAATAGATTTTCG
chicken-PCR-F	CTATAATCGATAATCCACGATTCA
chicken-PCR-R	CTTGACCTGTCTTATTAGCGAGG
duck-PCR-F	CATCTATCCTGCTAGCCGCC
duck-PCR-R	GGCTTGAGTGGAAGAATGCC

Table S2: LAMP amplification primers used in this study

Name		Sequences (5'-3')
LAMP-pig-1	F3	GCATGCATCATAGCCTTCA
	B3	CTCATATGGAAAGCATGAGG
	FIP	AACCTGCAATAGGGGCTTCTGTAAAAATACCTCTATACGGACTC
	BIP	GGAGGCTATGGCATAATGCGAATAATGGATAGGCTATGTAGTTTG
	LF	GGGCTTTTGGCAGTCAAAGATG
	LB	ACTATTCTAAACCCACTAA
LAMP-pig-2	F3	GCATGCATCATAGCCTTCA
	B3	GGTTATGATTATACCTCATATGGAA
	FIP	AACCTGCAATAGGGGCTTCTGTAAAAATACCTCTATACGGACTC
	BIP	ACTCGGAGGCTATGGCATAAGAATGGATAGGCTATGTAGTTTG
	LF	ACATGGGCTTTTGGCAGTCAAAG
	LB	TGCGAATCACTACTATTCTAAACCC
LAMP-ch-1	F3	CCCTAATCGGAGGCTGAA
	B3	CTTTGATTTGGGCTAGGGA
	FIP	ATTCATCCTAAATGGGAGATGGATGGGGCCTAAACCAAACACA
	BIP	AACCCACAACTCACTATTCTCACTAGGAATACAGTTGAGGTCAT
	LF	TTTGTGTTTGGTTTAGGCCCA
	LB	TCTCACCTTCATCCTCTACACAATT
LAMP-ch-2	F3	ACTCACCCTCCTAGCAAT
	B3	TTAGGACTTTGATTTGGGCTA
	FIP	AGATGGATGAGAAGGCTAGGATTTTCTCCTCCACCCTAATCGG
	BIP	AACCCACAACTCACTATTCTCACGGATAGGAATACAGTTGAGGTC
	LF	TCGTGTTTGTGTTTGGTTTAGGC
	LB	TTCATCCTCTACACAAT
LAMP-du-1	F3	CACCTCACGTGAAATCAGCA
	B3	CAGAGGCGCCAAAAAGCT
	FIP	AATGTGAGGAGGGCGAGGGGTGTCCGACGTGACTAGCT
	BIP	GGTTCACTCACCTCTCCTTGCCTGCCGCGATTACGCATTG
	LF	GGGAACGTATGGGCCTGA
	LB	GCATCTGTGGAATACTTCCACCA
LAMP-du-2	F3	CACCTCACGTGAAATCAGCA
	B3	TGGAGGATGCCGCGATT
	FIP	CGCAAAAATGTGAGGAGGGCGATCCGACGTGACTAGC

	TTCAG
BIP	GGTTCACTCACCTCTCCTTGCCCGCATTGAGATGGTGG AAGT
LF	GGGGTTTAGGGGGAACGT
LB	TTCAAAGTGGCATCTGTGGAAT

Table S3. ssDNA-reporters and crRNAs used in this study

Name	Sequences (5'-3')
T7-crRNA-F	TAATACGACTCACTATAGG
Pig-crRNA-1R	TTGAAGAACCAACATATATCAACCATCTACAACAGTAGAAAT
Pig-crRNA-2R	GAGAATATATACTTCTGGGTGTCCATCTACAACAGTAGAAAT
Pig-crRNA-3R	CTATGAAGGCTGTTGCTATAACGGATCTACAACAGTAGAAAT
Pig-crRNA-4R	CTATGAAGGCTGTTGCTATAACGGATCTACAACAGTAGAAAT
Pig-crRNA-5R	CAATAGTACTTGCAGCCGTACTGCATCTACAACAGTAGAAAT
Chicken-crRNA-1R	GGAGATAATTATAATTATTCATCCATCTACAACAGTAGAAAT
Chicken-crRNA-2R	TAAGTGGTTTGATGCGGTTGGCTTATCTACAACAGTAGAAAT
Chicken-crRNA-3R	GGATACTTGCATGTATATGTCTAGATCTACAACAGTAGAAAT
Chicken-crRNA-4R	GTGGATATGAGGCCCGGATTCATAATCTACAACAGTAGAAAT
Chicken-crRNA-5R	TGTAGTGAAGTTCATAATGAGTTGATCTACAACAGTAGAAAT
Duck-crRNA-1R	GCCCTGACCGAGGAACCAGAGGCGATCTACAACAGTAGAAAT
Duck-crRNA-2R	AAATAAAAGGAACCAGAGGCGCCAATCTACAACAGTAGAAAT
Duck-crRNA-3R	AAAATAAAAGGAACCAGAGGCGCCATCTACAACAGTAGAAAT
Duck-crRNA-4R	AAAAATAAAAGGAACCAGAGGCGCATCTACAACAGTAGAAAT
Duck-crRNA-5R	CCCTGACCGAGGAACCAGAGGCGCATCTACAACAGTAGAAAT
FAM-N12-Biotin	/5’-FAM/GTATCCAGTGCG/3’ Biotin /
JOE-dye	/5’-JOE/GTATCCAGTGCG/3’BHQ1/
Texas Red-dye	/5'-Texas Red /GTATCCAGTGCG/3’BHQ2/

Table S4. Species Information

Species	Breeds	GeneBank Accession No.
Pig	Chinese Wuzhishan	AF486867.1
	Chinese Meishan	AF304200.1
	Chinese Jinhua	KC469586.1
	Berkshire	KP765602.1
	Bamei	EF545583.1
	Tibetan	KM073256.1
	Rongchang	KM044239.1
	Hampshire	AY574046.1
	pietrain	KC469587.1
	Landrace	NC_000845.1
Chicken	Niya	MT635915.1
	Longsheng	MT635914.1
	Luhua	MT555049.1
	Qingyuan	MT635913.1
	Wanbei game	MT762345.1
	Huangshan black	MT762346.1
	Wuhua three-yellow	KM096864.1
	Zhengyang Yellow	KX987152.1
	Red jungle fowl	GU261708.1
	Tibetan	DQ648776.1
	Gushi	GU261678.1
Duck	Lin Wu	KJ794187.1
	Mallard	EU755253.1
	Jinding	MF069248.1
	Longsheng	KJ739616.1
	Xilin	KJ833586.1
	Sichuan	KX592536.1
	Pekin	EU755252.1
	Rongshui	KJ833587.1
	Shaoxing	HM010684.1
	Jianchang	FJ167857.1

Table S5. Simulate mixed meat products ingredients

Sample Number	Pig (mg)	Chicken (mg)	Duck (mg)	Cattle (mg)	Buffalo (mg)	Goat (mg)	Sheep (mg)
1	13.1	0	0	0	0	40.1	45
2	14.1	24.8	0	0	0	27.6	30
3	13.6	20	0	0	22	16.2	15.7
4	0	0	0	0	0	50	50
5	0	50	0	0	50	0	0
6	0	18	0	0	0	37.8	41.5
7	0	18	0	31.1	0	29.6	29.6
8	0	17	0	17.2	16.8	17.3	15.7
9	0	0	0	0	0	50	50
10	0	0	0	50	50	0	0
11	40.4	0	13	41.5	16.1	0	0
12	25.9	0	13.5	27.3	14.8	21.7	0
13	11	0	19.5	14.3	6.6	12.1	10
14	0	0	0	50	50	0	0
15	50	0	0	0	0	50	50
